# Using maternal and neonatal data collection systems for coronavirus disease 2019 (COVID-19) vaccines active safety surveillance in low- and middle-income countries: an international modified Delphi study

**DOI:** 10.12688/gatesopenres.13305.1

**Published:** 2021-07-12

**Authors:** Veronica Pingray, María Belizán, Sarah Matthews, Sabra Zaraa, Mabel Berrueta, Lisa M. Noguchi, Xu Xiong, Alejandra Gurtman, Judith Absalon, Jennifer C. Nelson, Lakshmi Panagiotakopoulos, Esperanca Sevene, Flor M. Munoz, Fernando Althabe, Kissa W. Mwamwitwa, Federico Rodriguez Cairoli, Steven A. Anderson, Elizabeth M. McClure, Christine Guillard, Annettee Nakimuli, Andy Stergachis, Pierre Buekens

**Affiliations:** 1 Institute for Clinical Effectiveness and Health Policy (IECS-CONICET), Ciudad de Buenos Aires, Buenos Aires, 1414, Argentina; 2 School of Public Health and Tropical Medicine, Tulane University, New Orleans, Louisiana, 70112, USA; 3 School of Pharmacy, University of Washington, Seattle, Washington, 98195, USA; 4 Jhpiego, Johns Hopkins University, Baltimore, Maryland, 21231, USA; 5 Vaccine Research and Development, Pfizer, Inc, Pearl River, New York, 10965, USA; 6 Kaiser Permanente, Washington Health Research Institute, Seattle, Washington, 98101, USA; 7 Centers for Disease Control and Prevention, Atlanta, Georgia, 30333, USA; 8 Department of Physiological Science, Clinical Pharmacology, Faculty of Medicine , Maputo, Mozambique, Eduardo Mondlane University/Manhiça Health Research Centre, Maputo, Maputo, 1102, Mozambique; 9 Departments of Pediatrics, Molecular Virology and Microbiology,, Baylor College of Medicine, Houston, Texas, 77004, USA; 10 UNDP-UNFPA-UNICEF-WHO-World Bank Special Programme of Research, Development and Research Training in Human Reproduction, Department of Sexual and Reproductive Health and Research, World Health Organization, Geneva, Geneva, 1211, Switzerland; 11 Tanzania Medicines and Medical Devices Authority, Dar es Salaam, Tanzania, 11000, Tanzania; 12 U. S. Food & Drug Administration, Silver Spring, Maryland, 20993, USA; 13 Social, Statistical and Environmental Sciences, Research Triangle Institute, Durham, North Carolina, 27709, USA; 14 World Health Organization, Geneva, Geneva, 1211, Switzerland; 15 Department of Obstetrics and Gynaecology, School of Medicine, Makerere University, Kampala, Kampala, 0000, Uganda; 16 School of Public Health, University of Washington, Seattle, Seattle, Washington, 98195, USA

**Keywords:** COVID-19 vaccine, pregnancy, Delphi Technique, active safety surveillance

## Abstract

**Background:** Given that pregnant women are now included among those for receipt coronavirus disease 2019 (COVID-19) vaccines, it is important to ensure that information systems can be used (or available) for active safety surveillance, especially in low- and middle-income countries (LMICs). The aim of this study was to build consensus about the use of existing maternal and neonatal data collection systems in LMICs for COVID-19 vaccines active safety surveillance, a basic set of variables, and the suitability and feasibility of including pregnant women and LMIC research networks in COVID-19 vaccines pre-licensure activities.

**Methods:** A three-stage modified Delphi study was conducted over three months in 2020. An international multidisciplinary panel of 16 experts participated. Ratings distributions and consensus were assessed, and ratings’ rationale was analyzed.

**Results:** The panel recommended using maternal and neonatal data collection systems for active safety surveillance in LMICs (median 9; disagreement index [DI] -0.92), but there was no consensus (median 6; DI 1.79) on the feasibility of adapting these systems. A basic set of 14 maternal, neonatal, and vaccination-related variables. Out of 16 experts, 11 supported a basic set of 14 maternal, neonatal, and vaccination-related variables for active safety surveillance. Seven experts agreed on a broader set of 26 variables. The inclusion of pregnant women for COVID-19 vaccines research (median 8; DI -0.61) was found appropriate, although there was uncertainty on its feasibility in terms of decision-makers’ acceptability (median 7; DI 10.00) and regulatory requirements (median 6; DI 0.51). There was no consensus (median 6; DI 2.35) on the feasibility of including research networks in LMICs for conducting clinical trials amongst pregnant women.

**Conclusions:** Although there was some uncertainty regarding feasibility, experts recommended using maternal and neonatal data collection systems and agreed on a common set of variables for COVID-19 vaccines active safety surveillance in LMICs.

## Introduction

The coronavirus disease 2019 (COVID-19) pandemic has created unprecedented global health challenges, triggering an accelerated development and distribution of safe and effective vaccines against severe acute respiratory syndrome coronavirus 2 (SARS-CoV-2) ^1^. The international research and development (R&D) effort in response to the enormous health burden associated with the COVID-19 pandemic is unprecedented in terms of scale and speed ^2, 3^. The World Health Organization (WHO) interim guidance for COVID-19 vaccines has recommended that pregnant women should be vaccinated against COVID-19 on the basis of a benefit vs risk assessment ^4, 5^. However, limited data from clinical trials is available on COVID-19 vaccine safety, immunogenicity, reactogenicity, and efficacy in pregnancy and their potential effects on the fetus or the neonate, particularly in LMICs ^4– 6^.

Pregnant women have been historically excluded from clinical trials for drugs and vaccines that do not target obstetric conditions ^7^. However, there is mounting evidence that pregnant women are at an increased risk for health complications from COVID-19 compared to non-pregnant women ^8, 9^. Excluding pregnant women from studies on vaccination in order to provide protection from theoretical risks is not warranted and can prevent this population from potentially beneficial inmunizations. 

There have been considerable international efforts to ensure equitable access to COVID-19 vaccines, including the COVAX Advance Market Commitment (AMC)'s efforts to ensure equitable access and distribution of COVID-19 vaccines to low-and-middle-income countries (LMIC) ^10^. Given these efforts to ensure LMIC access to vaccines and that pregnant women are now eligible to receive the COVID-19 vaccine, it will be essential to ensure that systems in LMICs can identify, evaluate, and respond to potential adverse events among pregnant women and their offspring. Following maternal immunization gaps in infrastructure, resources, training, data quality, and methods may limit establishment of effective pregnancy surveillance systems in LMICs ^11^. The accelerated vaccine development in the context of COVID-19 poses further considerations for resource-limited settings in LMICs, including the need to enhance site capacity, ensure regulatory approvals are met, and obtain vaccine decision-makers’ acceptance ^12^.

The importance of safety surveillance of these vaccines in pregnant women will be critical as pregnant women are vaccinated, especially for safety surveillance immediately post-licensure or post-emergency use authorization ^13^. It is in this timeframe where active safety surveillance must be conducted, as this is when safety concerns are likely to occur ^6^. Adverse events of special interest (AESI) for COVID-19 vaccines have been identified and obstetric AESIs have been considered for active monitoring ^14– 16^. After introducing a vaccine, it will be essential to utilize perinatal health information systems to monitor obstetric and neonatal outcomes. Identifying which variables will be necessary to monitor will be critical to ensure quality safety assessments. These efforts have already begun with the Global Alignment of Immunization Safety Assessment in pregnancy (GAIA) project and the WHO Global vaccine safety in pregnancy multi-country collaborative study (MCC) ^13, 17^. 

This study had two aims: 1) to build consensus about the use of existing maternal and neonatal data collection systems in LMICs for active safety surveillance of COVID-19 vaccines post-licensure, as well as the basic set of variables needed; and 2) to establish an expert consensus on the suitability and feasibility of including pregnant women and LMIC research networks in pre-licensure activities for novel COVID-19 vaccines.

## Methods

**Figure 1.**
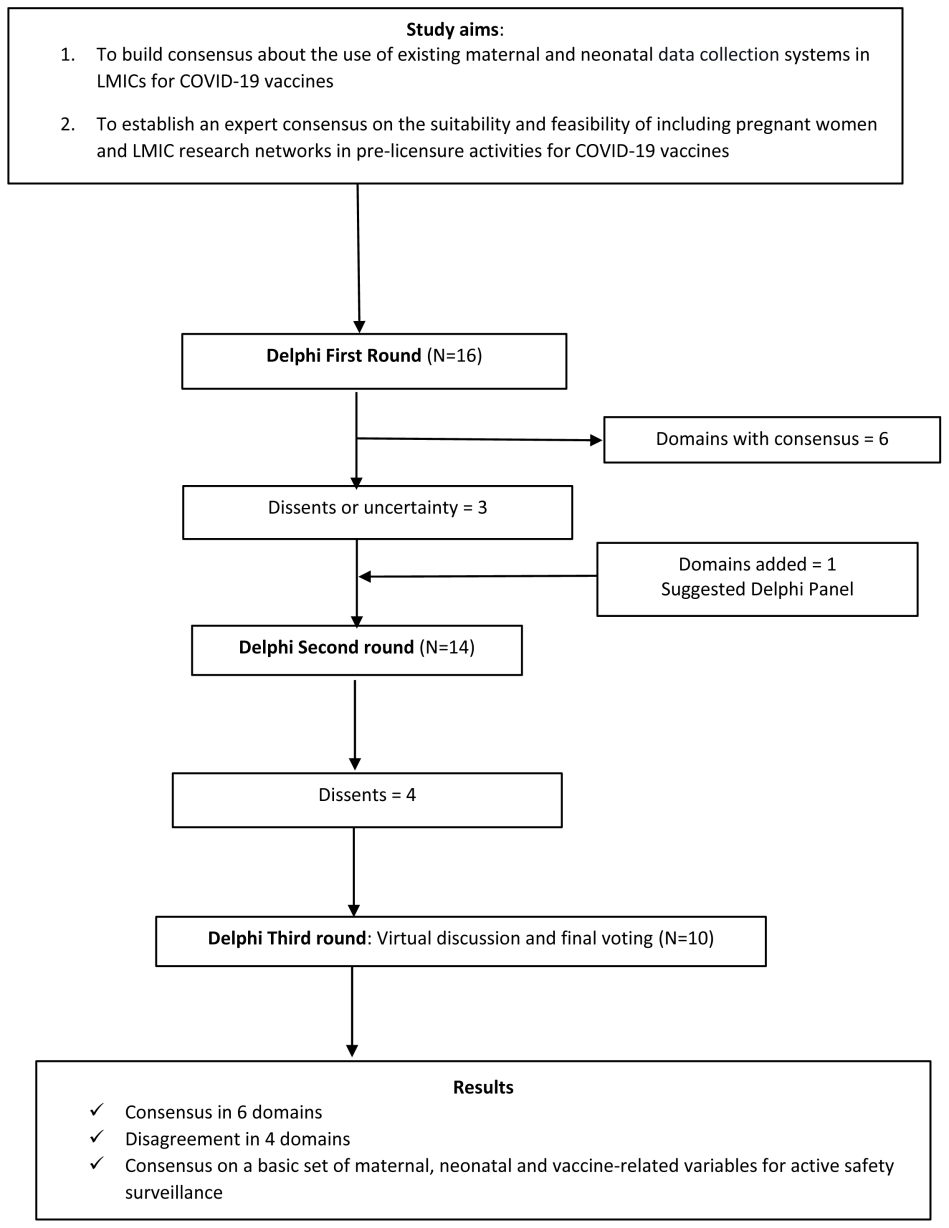
Flowchart of phases and procedures performed during the study. LMIC=low- and middle-income countries; COVID-19=coronavirus disease 2019.

A three-stage modified Delphi study was conducted between June 29 ^th^, 2020, and August 26 ^th^, 2020. This study first conducted two individual, online rounds and concluded with a third round: a virtual group discussion and final vote. Figure 1 outlines the study phases and procedures.

### Panel composition

This study is the second phase of a larger study entitled “ *Landscape analysis: Sentinel site readiness for Maternal Immunization Active Safety Surveillance in LMIC*” ( safeinpregnancy.org) that seeks to define the landscape for integrated maternal immunization active safety surveillance by reviewing the literature (phase 1), building consensus among experts (phase 2), and identifying potential sentinel sites in LMICs (phase 3). An independent Scientific and Technical Advisory Board (STAB) integrated by 12 experts in the field was already established to provide up-to-date and globally representative advice on the larger study. For the Delphi study, all members of the STAB and four additional experts identified by the Bill & Melinda Gates Foundation were nominated to integrate the panel. All the nominated experts had recognized international leadership in maternal immunization, vaccine safety, and maternal and neonatal health. During the recruitment process, as well as experts were selected based of their knowledge in maternal and perinatal health information systems for active safety surveillance. Geographical representation was another criteria taken into consideration. The rationale behind the 16-member panel was that they were large enough to permit diversity of representation while still being small enough to allow everyone to be involved in the group discussion.

To prevent the bias that could be introduced by knowing other leaders' opinions on the subject, the experts answered the questionnaires in each round anonymously and individually. The panel did not meet with each other face to face before the final meeting. The Delphi technique aims to generate consensus, and for this reason, each participant must know the opinion of the group. However, during all rounds, the shared data was de-identified, and in the final meeting, participants declared whether they had any conflict of interest and a moderator sought to prevent some participants from leading the discussion.

### Development of questionnaires

A bibliographic search was carried out in PubMed and grey literature. The following terms were employed to identify relevant peer-reviewed literature, reports published by governments or international health organizations (i.e. WHO), and other relevant articles to inform survey development: “COVID-19”, “coronavirus”, “SARS-CoV-2”, “pregnancy” and “pregnant women” were combined separately with the keywords: “active safety surveillance”, “vaccines”, “vaccine clinical trial”, “vaccine development”, “COVID-19 vaccine development”, “COVID-19 vaccine landscape”, “vaccine safety surveillance”, “sentinel site surveillance”, “information systems”, “safety monitoring”, “maternal immunization”, “low income countries”, “low- and middle-income countries” and “LMIC”.

This search identified publications that described factors affecting experts’ opinion on the use of routine information systems for COVID-19 vaccine active safety surveillance. Additionally, this search identified publications describing factors affecting experts' views on pregnant women's inclusion for novel COVID-19 vaccine R&D activities. Reported variables to be collected for maternal immunization safety monitoring were identified and listed. Subject matter experts were consulted to validate a preliminary list of variables based on the literature search. All this information was used to inform the development of the first questionnaire.

The first questionnaire included the following domains: a) appropriateness of using existing maternal and neonatal data collection systems for active safety surveillance, b) feasibility of adapting these systems prior to the pregnancy licensure of novel COVID-19 vaccines, c) benefits versus risks of including pregnant women in COVID-19 vaccine trials; d) feasibility of including pregnant women in the target population for COVID-19 vaccine R&D (in terms of the capacity of existing research networks and acceptability among vaccine decision-makers); and e) feasibility of incorporating existing research networks in LMICs (in terms of capacity and decision-makers’ acceptability). Additionally, lists of maternal, neonatal, and vaccine-related variables (i.e., name of the vaccine, date of vaccination, administration site, lot number) were included in the questionnaire to their prioritize their importance for active safety surveillance. The list of maternal and neonatal variables was selected from GAIA project case definitions piloted in 24 sentinel sites across four WHO regions ^17^. Questionnaires for the second and third rounds included a selection of questions to address disagreements that were noted in previous rounds. Questions related to selecting variables for active safety surveillance were re-evaluated in round two with new variables suggested by experts in the first round. All three questionnaires included both closed-ended and open-ended questions. The questionnaires can be found as extended data ^18^.

### Data collection

Data were collected through an iterative consensus exercise, starting with two rounds of online, individual surveys followed by a third round, which involved a virtual group discussion and final vote on remaining dissents. Online surveys were self-administered via Survey Monkey™. For both online rounds, panellists were asked to rate questions on a 9-point Likert RAND Appropriateness Scale (RAS) ^19^. In addition, the rationale for each rating was collected using open-ended questions.

During the second round, experts received the overall rating distributions and comments from the previous round and were asked to re-rate the questions that generated dissent.

The first survey was sent on June 29 ^th^, 2020, to 16 selected experts who had confirmed their interest and availability to participate. Three reminders were sent to participants with partial or no response over two weeks. The second survey was sent on July 22 ^nd^, 2020, to the same 16 experts, followed by four reminders over another two weeks. Completeness and consistency between the survey items were monitored during survey administration.

During the third round, experts met via virtual meeting on August 26 ^th^, 2020, to confirm the generated agreements and address remaining disagreements. Presentations and guided discussions were held in plenary sessions. The main questions to be discussed were developed by researchers with experience in qualitative research (MB, VP and SM). A meeting facilitator (PB) conducted and moderated the discussion (60 minutes). Detailed field notes were taken by one of the researchers (SM). During the plenary session, the system Poll Everywhere ^®^ was used to record the final individual vote. 

### Data analysis

Panel median rating and disagreement index (DI) were calculated to summarize experts’ ratings and measure consensus. The DI is a continuous scale used to measure panel ratings' dispersion, taken as an indicator of the level of agreement ^19^. A DI < 1 (including negative values) represents a complete agreement among panellists, while a DI ≥ 1 indicates disagreement among the panel. The RAND/UCLA appropriateness scale (i.e., RAND, a 9-point Likert scale ranging from 1 to 9) was used to classify domains as "appropriate", “inappropriate" or "uncertain", in accordance with: a) the median panel rating, and b) the DI. Domains with median ratings in the top third (score of 7–9) of the appropriateness scale were classified as “appropriate”, those in the bottom third were classified as “inappropriate” (score of 1–3), and those with intermediate median ratings were classified as “uncertain” (score of 4–6). Besides, domains with disagreement among the panel was also classified as “uncertain”. Table 1 provides the definitions for how each item was evaluated for “Appropriateness” and “Agreement”. The analysis was performed using STATA 14.0.

**Table 1.** Measure of disagreement index (DI) and RAND/UCLA appropriateness scale.

DI	Panel median rating								
	1	2	3	4	5	6	7	8	9
	Bottom third (1–3)			Intermediate third (4–6)			Top third (1–3)		
<1 (Agreement)	Inappropriate			Uncertain			Appropriate		
≥1 (Disagreement)									

Open-ended questions were analysed using a content analysis. Responses were coded by one researcher (SM) and reviewed by other researcher (MB), and matrices were developed to help interpret the findings. The analysis process was facilitated by a qualitative data management software Atlas.ti 8.4. All rationales that panellists provided to inform their quantitative responses are included in the findings, regardless of whether one or more person mentioned them.

The review was conducted and reported in line with the standards of the COREQ statement for qualitative studies and STROBE checklist for observational studies.

### Ethical approval

Approval was obtained from the Tulane University Internal Review Board (Approval number 2019–1453), United States, and the Comité de Ética en Investigación “Norberto Quirno”, Argentina (Approval number 1258/2019). All participants provided electronic written informed consent to participate in the study.

## Results

All 16 nominated experts from seven countries and three world regions participated in this consensus building exercise. Panel characteristics are described in Table 2. The multidisciplinary panel provided a diverse perspective of experts affiliated with different types of organizations (i.e., academic institutions, biopharmaceutical companies, government institutions, non-profit organizations, and international agencies). Most participants were working at institutions conducting projects related to COVID-19 vaccines (13/16). Sixteen experts completed Round 1, 14 completed Round 2, and ten experts from the same panel participated in the final virtual discussion and final vote.

**Table 2.** Panel characteristics.

	N = 16 (n)
**Gender**	
Female	13
Male	3
**Type of organization**	
Academia	7
Government organization	4
International Agency	2
Biopharmaceutical company	3
**Location**	
Region of the Americas	9
African Region	4
Europe	3
**Currently working on any activities relevant** **for COVID-19 immunization in pregnancy**	
Yes	13
No	3

Experts’ opinions on the following main themes related to COVID-19 vaccines were explored throughout the study: a) use and adaptation of existing maternal and neonatal data collection systems in LMICs for active safety surveillance; b) maternal, neonatal, and vaccine-related variables for active safety surveillance; c) inclusion of pregnant women in the target population for vaccine R&D; and d) inclusion of existing research networks from LMICs in pre-licensure vaccine R&D activities.

### Use and adaptation of existing maternal and neonatal data collection systems in LMICs for active safety surveillance

The expert panel agreed (RAND DI -0.92) on the appropriateness of using existing maternal and neonatal data collection **systems in LMICs** for active safety surveillance for COVID-19 vaccines, given that 12 out of 16 experts assigned a rating in the top third of the scale (7–9) and the panel median was 9 ( Table 3). This question reached a consensus in round 1 and was not re-evaluated in subsequent rounds.

*“There are a number of active information systems that could be employed to support these activities with minimal additional support.” (Round 1 response) *

Some participants who gave lower ratings on this item cited limitations in systems and their overall lack of existence. Some participants mentioned that existing systems would need to be adapted and improved to collect meaningful data and link maternal and neonatal information.

**Table 3.** Experts' ratings on the use of maternal and neonatal data collection systems in low- and middle-income countries (LMICs) for coronavirus disease 2019 (COVID-19) vaccines maternal and neonatal active safety surveillance.

	Maternal and neonatal active safety surveillance	
	Recommendation for the usage of existing information systems	Feasibility to adapt before licensure
***First-round (n=16)***		
**Rating distribution**		
Median (p25;p75)	9 (7;8,5)	7 (6,5;5,5)
**Agreement**		
RAND DI ^a^	-0.92	2.45
Agreement ^b^	Yes	**No**
Appropriateness ^c^	Appropriate	**Uncertain**
***Second round (n=14)***		
**Rating distribution**		
Median (p25;p75)	N/A	6 (5,25;7)
***Agreement***		
RAND DI ^a^	N/A	1,18
Agreement ^b^	N/A	**No**
Appropriateness ^c^	N/A	**Uncertain**
***Third round (n=10) ^d^***		
**Rating distribution**		
Median (p25;p75)	N/A	6 (3.5; 7)
**Agreement**		
RAND DI ^a^	N/A	1.79
Agreement ^b^	N/A	**No**
Appropriateness ^c^	N/A	**Uncertain**

References: ^a^ DI (disagreement index) is a measure that shows if there was wide or limited dispersion of panelist ratings. If the DI is ≥ 1, then it indicates 'extreme variation' in ratings. The lower the DI, the lower the level of disagreement; ^b^ Agreement was defined as DI <1; ^c^ Appropriateness considers median scores and the level of disagreement. Items with median scores in the 1–3 range are classified as inappropriate, those in the 4–6 range as uncertain, and those in the 7–9 range as appropriate. However, all items rated "with disagreement," whatever the median, are classified as uncertain. N/A: not applicable

The panel consistently disagreed (DI 2.45; 1.18; 1.79 for rounds 1 through 3, respectively) on the feasibility of existing maternal and neonatal data collection systems in LMICs to incorporate new changes before approval or licensure of novel COVID-19 vaccines so that adequate **active safety surveillance** can be performed amongst pregnant women ( Table 3). This indicates a considerable level of experts’ uncertainty regarding this feasibility. Experts acknowledged that there are existing systems that could be modified. However, the variability between systems and locations was noted, and the feasibility of adapting these systems depended on the financial and human resources needed to make these changes before licensure. Support would be required to increase technical, managerial, and staff capacity. Given the accelerated timeline of COVID-19 vaccine development, it was noted that there might not be enough time or resources to adapt these systems before vaccine approval or deployment ( Box 1).

**Box 1. Experts' opinion on tailoring existing maternal and neonatal data collection systems for active safety surveillance of coronavirus disease 2019 (COVID-19) vaccines amongst pregnant women**

**From a panelist point of view** 

- **It is appropriate** to use existing Maternal, Newborn and Child Health (MNCH)information systems in LMICs for active safety surveillance of a novel COVID-19 vaccine licensed for usage amongst pregnant women.
- **It is reasonable and desirable to adapt existing systems**, and there is a clear opportunity to leverage existing MNCH information systems in LMICs for active safety surveillance of COVID-19 vaccines in pregnant women.
- Existing systems **would provide background rates of outcomes prior to vaccine introduction**. There are sentinel sites and demographic health survey sites ready to start baseline measurements of critical data for active safety surveillance.
- **In the COVID-19 vaccine accelerated timeline, there may not be time to adapt systems**.
- **Existing systems would need to be adapted and improved** to collect meaningful data and link maternal and infant information. Systems could potentially augment active safety surveillance, and this could be significantly improved with targeted support.
- **Merging existing MNCH systems with a pregnancy registry** would be needed to optimize surveillance data for maternal immunization programs.
- **Uncertainties on the feasibility to adapt existing systems** prior to vaccine licensure.
  Feasibility would be conditioned by:
  - Variability of the systems in different countries;
  - Financial resources allocated to the requested modifications:
  - Availability of support to increase technical, managerial, and staff capacity;
  - Policymaker approvals and funds.

*“Some systems are updated very irregularly due to funding limitations. With financial support, this could be overcome. However, financial support might need to include training of clinical staff, as well as additional staff responsible for QA/QC (quality assessment and quality control) of data, etc. - this is highly dependent on the country and system in question.” (Round 1 response)*
*“The incorporation would vary widely between LMICs and even within countries, depending on the available resources to do so. Overall, this would be a much slower process that would frustrate the people planning for the surveillance”. (Round 2 response)*
*“Timeliness is likely to be an issue given that a COVID-19 vaccine is needed on an accelerated timeline” (Round 3 response)*

### Maternal, neonatal, and vaccine variables for active safety surveillance

Table 4 shows the final set of 26 maternal, neonatal and vaccine-related variables prioritized by the experts. Variables were prioritized if they were selected by at least seven panel members (50% of the panel) during the second round.

**Table 4.** Prioritized maternal, neonatal, and vaccine-related variables for coronavirus disease 2019 (COVID-19) vaccines active safety surveillance.

Variable (votes) N=14		
*Maternal*	*Neonatal outcomes*	*Vaccine-related variables*
**Maternal death (14)**	**Neonatal death (14)**	**Date of vaccination (14)**
**Spontaneous abortion (12)**	**Preterm birth (14)**	**Vaccine lot number (13)**
**Intrauterine growth retardation (11)**	**Stillbirth (13)**	**Name of vaccination (13)**
Hypertensive disorders of pregnancy (9)	**Congenital anomalies (13)**	**Gestational age at vaccination (11)**
Threatened preterm labor (8)	**Low birth weight (13)**	Dosage (9)
Maternal pneumonia (8)	**Small for gestational age (12)**	Vaccine expiration date (7)
Postpartum hemorrhage (7)	**Respiratory distress (11)**	
Maternal age (7)	Live birth (9)	
Chorioamnionitis (7)	Neurodevelopmental delay (7)	
	Microcephaly (7)	
	***Enabling term***	
	Assessment of gestational age (10)	

Note: variables prioritized by 80% of the experts' panel are highlighted in bold.

The following nine **maternal** variables were prioritized after receiving the vote of at least half of the experts' panel: maternal death, spontaneous abortion, hypertensive disorders of pregnancy, intrauterine growth retardation, threatened preterm labor, maternal age, maternal pneumonia, postpartum hemorrhage, and chorioamnionitis. All variables were prioritized during both rounds, except for postpartum hemorrhage, maternal age, and pneumonia, which were only prioritized during the second round. The following maternal variables were voted less often (by 1 to 6 experts): antenatal bleeding, postpartum hemorrhage, gestational diabetes, history of asthma or other respiratory disorders, endometritis, anemia, maternal gestational weight gain, hyperemesis gravidarum, dysfunctional labor, abnormal placentation, and pathways to preterm birth.

For the **neonatal** (late fetal period or neonatal) variables, at least half of the experts prioritized the following 11 variables (10 medical conditions and 1 enabling term): neonatal death, preterm birth, stillbirth, congenital anomalies, low birth weight, small for gestational age, respiratory distress, live birth, neurodevelopmental delay, microcephaly and assessment of gestational age. Other neonatal variables were prioritized less frequently: failure to thrive, neonatal encephalopathy, and neonatal seizures. All neonatal variables had very similar frequency distributions in each round.

At least half of the experts prioritized the following six **vaccine-related** variables were the following: date of vaccination, vaccine lot number, name of the vaccine, dosage, vaccine expiration date and gestational age at vaccination. All these variables were prioritized on both rounds. Other vaccine-related variables less frequently prioritized were: time of reconstitution, time of vaccination, manufacture date, and diluent expiration date. Similar to the neonatal variables, vaccine-related variables presented very similar frequency distributions in each round.

A smaller set of 14 variables were identified by increasing the expert panel's level of agreement to 80%. In this case, the variables that are prioritized were those that received at least 11 votes and are as follows: ****maternal**** - maternal death, spontaneous abortion, intrauterine growth retardation; ****neonatal**** - neonatal death, preterm birth, stillbirth, congenital anomalies, low birth weight, small for gestational age, respiratory distress; and ****vaccine-related**** - date of vaccination, vaccine lot number, name of the vaccine, gestational age at vaccination.

### Inclusion of pregnant women in the target population for COVID-19 vaccine R&D

The **suitability** of the inclusion of pregnant women in the target population for novel COVID-19 vaccine R&D activities was evaluated according to two factors: “Potential benefits versus the potential risks” and “Time appropriateness,” and the results are presented in Table 5. The panel weakly agreed (RAND DI -3.08) that the **potential** **benefits outweigh the potential risks** of including pregnant women for COVID-19 vaccine R&D and found this statement appropriate given that the panel median rating was 7 (top third of scale: 7–9). However, six members still reported uncertainty, as indicated by ratings in the intermediate third of the scale (4–6), and one expert considered the risks to outweigh the benefits, as indicated by their reported rating in the bottom third (1–3). The panel agreed (RAND DI -0.93) that it is an **appropriate time** in the COVID-19 vaccine R&D landscape to consider pregnant women for inclusion in the target population, given that 12/16 experts assigned a rating in the top third of the scale (7–9) and the panel median was 9. These two questions reached consensus in Round 1 of this Delphi study and were not re-rated in subsequent rounds. From experts’ comments, it was observed that ratings on suitability reflected their perception of what recent maternal immunization advances have had on maternal and neonatal health. Some experts viewed that this field has advanced to the point that pregnant women should be included to facilitate decision-making for immunization policy.

**Table 5.** Experts' ratings on the inclusion of pregnant women in the target population for coronavirus disease 2019 (COVID-19) vaccine research and development (R&D) activities.

	Suitability		Feasibility			General recommendation for inclusion

	Risks versus benefits	Time appropriateness	Decision-makers’ acceptability	Capacity of research networks	Regulatory requirements	
***First-round (n=16)***						
**Rating distribution**						
Median (p25;p75)	7 (6;8)	9 (7;9)	6 (4;7)	8 (7;8)	N/A	8 (7.5;8.5)
**Agreement**						
RAND DI ^a^	-0,71	-0.93	1.73	-0.71	N/A	-0.61
Agreement ^b^	Yes	Yes	**No**	Yes	N/A	Yes
Appropriateness ^c^	Appropriate	Appropriate	**Uncertain**	Appropriate	N/A	Appropriate
***Second round (n=14)***						
**Rating distribution**						
Median (p25;p75)	N/A	N/A	6.5 (4.25;7)	N/A	6.5 (3.25;7)	N/A
***Agreement***						
RAND DI ^a^	N/A	N/A	2.04	N/A	1.77	N/A
Agreement ^b^	N/A	N/A	**No**	N/A	**No**	N/A
Appropriateness ^c^	N/A	N/A	**Uncertain**	N/A	**Uncertain**	N/A
***Third round (n=10) ^d^***						
**Rating distribution**						
Median (p25;p75)	N/A	N/A	7 (6;7.25)	N/A	6 (5.25; 6)	N/A
**Agreement**						
RAND DI ^a^	N/A	N/A	10.00	N/A	0.51	N/A
Agreement ^b^	N/A	N/A	**No**	N/A	Yes	N/A
Appropriateness ^c^	N/A	N/A	**Uncertain**	N/A	**Uncertain**	N/A

References: ^a^ DI (disagreement index) is a measure that shows if there was wide or limited dispersion of panelist ratings. If the DI is ≥ 1, then it indicates 'extreme variation' in ratings. The lower the DI, the lower the level of disagreement (i.e., the higher the level of agreement/ better consensus).; ^b^ Agreement was defined as DI <1; ^c^ Appropriateness considers median scores and the level of disagreement. Items with median scores in the 1–3 range are classified as inappropriate, those in the 4–6 range as uncertain, and those in the 7–9 range as appropriate. However, all items rated "with disagreement," whatever the median, are classified as uncertain. N/A: not applicable

Experts' perceptions on the existing evidence that would consider pregnant women an at-risk population for risk of COVID-19 on pregnancy and subsequent consequences to the fetus varied. For some experts that positively support the inclusion of pregnant women in novel COVID-19 R&D activities, this population's exclusion is a violation of pregnant women’s rights and may result in vaccine hesitancy.

*“Leaving out pregnant women may result in a vaccine limited to use in non-pregnant women, omitting a large portion of reproductive age women from potential vaccine benefit. A novel COVID-19 vaccine that is branded as not for pregnant women (due to unknown risk in pregnancy) increases the risk of the vaccine being viewed with suspicion and fear. Leaving pregnant women out of the R&D program is patriarchal and morally unjustified.” (Round 1 response)*

**The feasibility** of including pregnant women in the target population for novel COVID-19 vaccine R&D activities was evaluated according to two factors: “Vaccine R&D decision-makers’ acceptability” and “Capacity of existing research networks”. Experts were also provided the opportunity to suggest other factors that may hinder the feasibility. After analysing open-ended responses from round 1, another factor related to feasibility was identified and evaluated in Round 2: “Regulatory requirements of maternal immunization vaccine studies”. There was no consensus throughout all three rounds (RAND DI 1.73; 2.04; 10.00; for rounds 1 through 3 respectively) regarding **decision-makers' acceptability**. This indicates a considerable level of expert uncertainty on vaccine R&D decision-makers’ acceptability of including pregnant women in the target population for novel COVID-19 vaccine R&D activities. From the experts’ point of view, decision-makers' acceptability may be conditional on regional differences, liability issues, interactions among decision-makers, and the influence certain groups have on each other, politics, and/or access to the vaccine. The necessity to develop and license a novel COVID-19 vaccine quickly to address the ongoing pandemic was considered a barrier to include pregnant women.

*“Given the urgency for R&D in COVID-19, it may be difficult to obtain the necessary regulatory/ethics approvals for inclusion of pregnant women within the timeframe”. (Round 2 response)*

Another barrier outlined was that vaccine developers might not want to take the risk of including this population in vaccine R&D activities when they are utilizing novel vaccine platforms.

*“Even though its is recommended by regulators to include pregnant women in clinical trials for COVID19 vaccine, vaccine developers might not want to take the risk of involving pregnant women particularly for vaccines using new platforms. This might be sorted out with trials conducted by government agencies”. (Round 2 response) *

One facilitator contributing to the acceptability of vaccine developers and funding agencies mentioned by some respondents was that regulatory agencies are requesting vaccine developers consider the inclusion of diverse populations, including pregnant women, in their pre-licensure studies.

How the pandemic has progressed in certain regions may also affect the acceptability of vaccine decision-makers. For example, countries that were significantly affected in the early stages of the pandemic may have more of a priority to include pregnant women, increasing acceptability of vaccine decision-makers. Differences in acceptability of including this population can be seen between regions with their previous implementation of maternal vaccines (e.g., flu and Tdap), where it was easier to implement in some regions versus others.

*“There are differences and that this can be seen with the implementation of previous vaccines (e.g. flu and Tdap), where it was easier to implement this in some regions versus others.” (Round 3 response)*

During the virtual meeting, panelists pointed out that the region where they currently work (their local working setting) may influence their responses. Given the large variety of decision-makers, their answers may have been influenced by which decision-makers they were most familiar with. Acceptability among academics and medical organizations may be higher, but there is a dependence on those “higher-up” in authority to make the decision.

*“Politics and access to the vaccine influence this as well. Although acceptability among academics and medical organizations may be higher, there is a dependence on those “higher-up” to make this decision to implement the vaccine.” (Round 3 response)*

The panel agreed (RAND DI -0.71) and found appropriate the **capacity of existing research networks** for including pregnant women for COVID-19 vaccine research, as shown by a panel median of 8 and that the majority of experts (13 out of 16) provided a rating within the top third of the rating scale (7–9). This question reached consensus in round 1 and was not re-evaluated in subsequent rounds. Experts mentioned in the open-ended question responses and agreed on existing networks' capacity to include pregnant women and that they would need minimal support. The panel agreed (RAND DI -0.61) and found appropriate the **recommendation to include pregnant women** in the target population for novel COVID-19 vaccine R&D activities, indicated by 12 out of 16 experts assigning a rating between 7–9 and the panel median was 8 (top third of the appropriateness scale). Despite this strong recommendation, in the open-ended question, some experts pointed out that it is essential to have better evidence about the burden of the disease that COVID-19 has on pregnant women and neonates or to conduct developmental and reproductive toxicology (DART) studies before including pregnant women in this target population. For some experts that highly recommend the inclusion of pregnant women in novel COVID-19 R&D activities, it is an ethical issue regarding women’s rights to access to vaccines.

“If pregnant women are not included in the COVID-19 vaccine R&D activities, then they will not be beneficiaries of the vaccines when licensed, which would unfortunate to exclude such a a relatively high risk group.”

They noted that in LMICs, many health workers and front-line caretakers are women of reproductive age. Therefore, the inclusion of pregnant women in trials would allow them access to the vaccine for those health workers.

*“Pregnant women deserve access to COVID-19 vaccines; moreover, many frontline workers at high risk of COVID disease may be pregnant. If you don't have a vaccine that you can give to pregnant women, you don't have a vaccine for frontline workers”. (Round 1 response)*

### Including existing research networks from LMICs in COVID-19 R&D activities amongst pregnant women

During the first round, panelists agreed (RAND DI 1.73) that there is uncertainty (panel median 5.5 – intermediate third of rating scale) on the **acceptability among decision-makers of incorporating existing research networks in LMICs**. During the subsequent rounds 2 and 3, the panel continued to show disagreement (RAND DI 2.04; 2.35) on their opinion regarding this domain ( Table 6). In the open-ended questions and during the virtual meeting plenary discussion, panelists mentioned that decision-makers’ acceptability would be determined by the specific network's demonstrated capacity (e.g., capacity to properly care for study participants, ability to document the outcomes of interest or test the hypotheses under evaluation). Consequently, it would influence how much needs to be invested in these networks to ensure sufficient capacity to conduct this work. They also highlighted the potential ethical concerns that may affect decision-makers’ acceptability due to concerns about unethical clinical research among LMIC populations in the past.

**Table 6.** Experts' ratings on the inclusion of research networks from low- and middle-income countries (LMICs) in coronavirus disease 2019 (COVID-19) vaccine pre-licensure activities amongst pregnant women.

	Pre-licensure R&D activities	
	Decision-makers’ acceptability	Capacity of existing research networks
***First-round (n=16)***		
**Rating distribution**		
Median (p25;p75)	5.5 (4;6)	7 (6.5;8)
**Agreement**		
RAND DI ^a^	0.63	-0.68
Agreement ^b^	Yes	Yes
Appropriateness ^c^	**Uncertain**	Appropriate
***Second round (n=14)***		
**Rating distribution**		
Median (p25;p75)	6 (5.25;7)	N/A N/A N/A N/A
***Agreement***		
RAND DI ^a^	3.16	
Agreement ^b^	**No**	
Appropriateness ^c^	**Uncertain**	
***Third round (n=10) ^d^***		
**Rating distribution**		
Median (p25;p75)	6 (5; 7)	N/A
**Agreement**		
RAND DI ^a^	2.35	N/A
Agreement ^b^	**No**	N/A
Appropriateness ^c^	**Uncertain**	N/A

References: ^a^ DI (disagreement index) is a measure that shows if there was wide or limited dispersion of panelist ratings. If the DI is ≥ 1, then it indicates 'extreme variation' in ratings. The lower the DI, the lower the level of disagreement (i.e., the higher the level of agreement/ better consensus).; ^b^ Agreement was defined as DI <1; ^c^ Appropriateness considers median scores and the level of disagreement. Items with median scores in the 1–3 range are classified as inappropriate, those in the 4–6 range as uncertain, and those in the 7–9 range as appropriate. However, all items rated "with disagreement," whatever the median, are classified as uncertain. N/A: not applicable; R&D: research and development.

*“Would need to have sufficient evidence on the burden and risk of serious disease in pregnant women to ensure acceptability of including LMICs research network, ....would Would also need to demonstrate that long term follow up is possible in those settings” (Round 2 response) *

In contrast to the previous domain, the panel agreed (RAND DI -0.68) on the **capacity of existing research networks in LMICs** to participate in maternal COVID-19 vaccine R&D, given that 12 out of 16 experts assigned a rating between 7–9 and the panel median of 7 (top third of rating scale: 7–9). This question reached a consensus in the first round and was not re-evaluated in subsequent rounds. In the open-ended questions and during the virtual meeting, panelists remarked that capacity is dependent on the network and location as there is variability between networks in LMICs. Some experts commented that it might be necessary to conduct this work in high-income countries with greater capacity concomitantly or before utilizing networks in LMICs.

## Discussion

### Main findings

In the present study, a systematic approach was used to explore experts’ opinions on the use of existing maternal and neonatal data collection systems and a list of prioritized variables for COVID-19 active safety surveillance, as well as the inclusion of pregnant women and LMICs research network in COVID-19 R&D. A diverse international multidisciplinary panel recommended using maternal and neonatal data collection systems for active safety surveillance in LMICs. There was some uncertainty as to the feasibility of adapting these systems in a timely manner given the accelerated timeline of COVID-19 vaccine development. For active safety surveillance, at least half of the experts supported the prioritization of nine maternal, eleven neonatal, and six vaccine-related variables. The experts found suitable the inclusion of pregnant women amongst the target population for COVID-19 vaccines R&D, although there is uncertainty on its feasibility. Similarly, there is uncertainty and no consensus on the feasibility of including research networks in LMICs for conducting pre-licensure activities amongst pregnant women.

### Interpretation

Experts highlighted the importance of including LMICs in pre- and post-licensure/post-authorization activities for COVID-19 maternal immunization, but various disagreements have highlighted specific barriers. In their interim guidance recommendation, the WHO recommends the COVID-19 vaccine for pregnant women based on a benefit vs risk assessment. Given this, the next steps will be to monitor the safety of these vaccines in pregnant women and their potential effects on the fetus and the neonate ^6^. To ensure generalizability to LMICs, it is essential to engage them in these R&D and active safety surveillance activities ^20^. Insights from this panel can help guide the next steps to engage LMICs following licensure of COVID-19 vaccines and echo the vaccine community's opinion on the necessity of these activities.

This panel reached a consensus about the capacity of LMIC research networks to be included in R&D activities and their recommendation on the usage of existing LMIC information systems for maternal and neonatal vaccine active safety surveillance of the novel COVID-19 vaccine. However, it was a commonly cited topic for both pre-licensure and post-licensure/post-authorization activities that the feasibility of including LMICs in these activities would require investment in time, resources and improving decision-makers acceptability ^17, 21, 22^. Studies of feasibility for multi-country active safety surveillance systems in LMICs noted that an initial investment in capacity is required for such activities ^17^, which may reflect the panel’s uncertainty in feasibility given that these activities require time. Investment in capacity involves the engagement of vaccine decision-makers. This panel's dissent regarding decision-makers' acceptability on inclusion and comments regarding the feasibility of adapting information systems for active safety surveillance reflect the need to engage stakeholders. To increase acceptability and prioritization of maternal immunization efforts, decision-makers at the global, regional, national, and local levels must be involved in vaccine R&D activities, and their varying baseline awareness and perceptions of maternal immunization understood ^23^. The panel’s dissent suggests that decision-makers lack of acceptability may pose the most significant barrier to COVID-19 R&D vaccine activities in LMICs. The influence of their awareness, priority, and investment in these activities has been cited as a barrier ^24, 25^. Insights from previous pandemics have highlighted that there is a need for solutions for liability and ethical concerns and promotion of R&D activities to engage decision-makers ^21, 25^. Experts commented that decision-makers have concerns regarding the inclusion of pregnant women for similar reasons. These barriers should be considered and addressed to ensure the timely inclusion of LMICs.

Given the WHO’s interim guidance on use of the authorized COVID-19 vaccines during pregnancy ^4, 5^, it is key to act quickly and utilize already existing systems so that quality data on vaccine safety in pregnancy can be collected from LMICs. The introduction of maternal vaccinations must occur almost concurrently across the globe in the high-, middle- and low-income countries, which requires building upon existing resources and capacity of systems in LMICs ^26^. This panel has reached a consensus regarding maternal and neonatal data collection systems usage in LMICs for maternal and neonatal active safety surveillance for COVID-19. Efforts to ensure access to COVID-19 vaccines in LMICs are already underway ^10^. Experts highlighted the existence of systems that could be strengthened and adapted for vaccine surveillance, reflecting what other publications have also stated ^22, 27^. The adaptation of these systems for COVID-19 vaccine active safety surveillance is thus a critical next step to ensuring the collection of quality vaccine safety data.

Despite the recommendation of using these existing systems, the investment and necessary adaptation in these systems may be a challenge to collecting high quality vaccine safety data in LMICs. Experts were uncertain about the feasibility of adapting existing LMIC systems on time for COVID-19 maternal immunization. Along with other authors, this panel has cited that resources and investment are needed to adapt these systems ^13, 28^. The urgency to adapt in time has posed a barrier to the establishment of post vaccination maternal and neonatal surveillance systems during COVID-19 pandemic and other outbreaks, such as Ebola ^25^. To address these barriers, it will be critical to plan and act quickly and utilize and leverage already existing systems so that quality data on vaccine safety in pregnancy can be collected from LMICs.

This panel’s prioritization of maternal and neonatal outcomes for vaccine active safety surveillance for COVID-19 provided interim, expert insight. According to the WHO's safety surveillance manual for COVID-19 vaccines, the outcomes specifically mentioned for monitoring adverse events in pregnancy are maternal mortality, stillbirth, miscarriage, neonatal mortality, and congenital anomalies. These outlined outcomes were also prioritized by the experts’ in this Delphi study. The GAIA project has created 25 standardized case definitions for maternal and neonatal outcomes ^13^. With the expert insight on the prioritization of these variables given here, a minimal set of maternal, neonatal, and vaccine-related variables necessary for COVID-19 active safety surveillance can be inferred. Besides, this panel's prioritization of maternal, neonatal, and vaccine-related variables can guide the required adaptation of existing maternal and neonatal data collection systems in LMICs for COVID-19 active safety surveillance.

There was established consensus on the need to include pregnant women in the target population for vaccine R&D, and the expert panelists agreed that the potential benefits outweigh the risks which is supported by recent evidence on the severity of disease and DART studies. 

The panel agreed that now is an appropriate time in the COVID-19 vaccine R&D landscape to consider including pregnant women in the target population. These findings align with the Institute of Medicine report that recommended that pregnant women be deemed eligible to participate in clinical trials and the WHO interim guidance for the approved vaccines ^4, 5, 29^. The ambiguous existing regulations concerning whether and when pregnant women should be included in clinical trials remains one of the main reasons for the persistent underrepresentation of this population in research ^30^.

The panel's dissent over the feasibility of the inclusion of pregnant women in the target population for novel COVID-19 vaccine R&D activities is also concordant with the literature. The concern of the panel was mainly about including pregnant women in early clinical studies to be cautious during a time of high uncertainty with little data available. Exclusionary criteria imposed by IRBs, federal guidelines, and codes of regulation have further contributed to this challenge ^31– 33^. This emerging consensus that systematic exclusion of pregnant women from study involvement ultimately leaves these women and their fetuses susceptible to potentially substantially higher harm due to the lack of rigorous clinical data on the safety of drugs and other treatments have pushed clinicians, academics, bioethicists, and professional societies to increasingly call for a re-examination of the routine procedure of excluding pregnant women from clinical research in recent years ^34^. This panel’s dissent regarding the effect of regulatory requirements on the feasibility of including pregnant women in the target population for COVID-19 vaccine R&D activities suggests that additional guidance and regulation may be required to assist IRBs and research sponsors move toward an equitable, ethical, and adequately protected inclusion of pregnant women in vaccine R&D in the context of COVID-19.

### Strengths and limitations

The study has several strengths. First, a methodologically rigorous, transparent, and reproducible process guided the development of this consensus study. Second, the methodology was defined and prior to the study commencing, and it was based on an extensively accepted consensus method technique: the modified-Delphi method. Third, a diverse panel affiliated with a broad spectrum of organizations and who were geographically distant participated in this study. Finally, the questionnaire included both closed- and open-ended questions, providing rich insight to experts’ ratings with qualitative data.

However, there were limitations to this study. First, inherent to any consensus process, there is some level of bias due to the influence of interpersonal dynamics. We tried to mitigate this by conducting two individualized online rounds and asking participants for disclosure of interest. Second, the small panel size did not allow for a subgroup analysis. It would have been particularly interesting to explore experts’ views by the type of country they were representing (high income countries vs. LMICs). Third, a classic in-person meeting was not possible given travel restrictions during the pandemic, so a virtual meeting was conducted. A guided discussion with prompts and probes was developed to encourage and facilitate the discussion to overcome this limitation. Lastly, given time-constraints to report findings, pre-testing and pilot questionnaires were omitted. We tried to ameliorate this by asking five experts in immunizations or maternal health who did not participate in the study to assess the questionnaires' content, their clarity, and the relevance of the included items.

## Conclusion

A core set of variables was identified for COVID-19 vaccine maternal and neonatal active safety surveillance. More complex discussions may need to be undertaken to better understand the barriers and strategies for using maternal and neonatal data collection systems in LMICs for post-licensure activities. Further work will also be required to generate consensus- on the inclusion of pregnant women and research networks from LMICs, as safety data on COVID-19 vaccines become available. 

## Data availability

### Underlying data

Open Science Framework: Using maternal and neonatal data collection systems for coronavirus disease 2019 (COVID-19) vaccines active safety surveillance in low- and middle-income countries: an international modified Delphi study - Supplementary materials. https://doi.org/10.17605/OSF.IO/ZNS2D ^18^.

This project contains the following underlying data:

• Active Safety Surveillance for COVID19 Dataset Round 1.csv
• Active Safety Surveillance for COVID19 Dataset Round 2.csv
• Active Safety Surveillance for COVID19 Dataset Round 3.csv
• Minutes Active Safety Surveillance in LMICs.docx

### Extended data

Open Science Framework: Using maternal and neonatal data collection systems for coronavirus disease 2019 (COVID-19) vaccines active safety surveillance in low- and middle-income countries: an international modified Delphi study - Supplementary materials. https://doi.org/10.17605/OSF.IO/ZNS2D ^18^.

This project contains the following extended data:

• Delphi Methods.pdf
• Delphi Results.pdf
• Discussion.pdf
• Supplementary materials_survey_methods.docx

Extended data are available under the terms of the Creative Commons Zero "No rights reserved" data waiver (CC0 1.0 Public domain dedication).

## References

[1] Thanh Le T Andreadakis Z Kumar A : The COVID-19 vaccine development landscape. *Nat Rev Drug Discov.* 2020;19(5):305–306. 10.1038/d41573-020-00073-5 32273591

[2] Lurie N Saville M Hatchett R : Developing Covid-19 Vaccines at Pandemic Speed. *N Engl J Med.* 2020;382(21):1969–1973. 10.1056/NEJMp2005630 32227757

[3] Berkley S : COVID-19 needs a big science approach. *Science.* 2020;367(6485):eabb8654. 10.1126/science.abb8654 32213646

[4] World Health Organization: Interim recommendations for use of the Moderna mRNA-1273 vaccine against COVID-19: interim guidance, 25 January 2021.2021. Reference Source

[5] World Health Organization: Coronavirus disease (COVID-19): Vaccines safety.2021. Reference Source 37184163

[6] Klein SL Creisher PS Burd I : COVID-19 vaccine testing in pregnant females is necessary. *J Clin Invest.* 2021;131(5):e147553. 10.1172/JCI147553 33444286 PMC7919709

[7] Schwartz DA Graham AL : Potential Maternal and Infant Outcomes from (Wuhan) Coronavirus 2019-nCoV Infecting Pregnant Women: Lessons from SARS, MERS, and Other Human Coronavirus Infections. *Viruses.* 2020;12(2):194. 10.3390/v12020194 32050635 PMC7077337

[8] Delahoy MJ Whitaker M O’Halloran A : Characteristics and maternal and birth outcomes of hospitalized pregnant women with laboratory-confirmed COVID-19 - COVID-NET, 13 States, March 1-August 22, 2020. *MMWR Morb Mortal Wkly Rep.* 2020;69(38):1347–1354. 10.15585/mmwr.mm6938e1 32970655 PMC7727497

[9] Adhikarla H Montoya M Jr Nery N : Kinetics of Anti-Zika IgG Antibodies During Follow-up of Infants Exposed to Zika Virus in Utero.Amer Soc Trop Med & Hygiene 8000 Westpark Dr, Ste 130, McLean, VA 22101 USA;2018;522–522.

[10] Gavi TVA: The Gavi COVAX AMC: An investment opportunity.2020. Reference Source

[11] Lackritz E Stepanchak M Stergachis A : Maternal immunization safety monitoring in low-and middle-income countries: a roadmap for program development.Bill & Melinda Gates Foundation and Global Alliance to Prevent Prematurity and Stillbirth (GAPPS),2017. Reference Source

[12] COVID-19 Clinical Research Coalition. Electronic address: nick.white@covid19crc.org: Global coalition to accelerate COVID-19 clinical research in resource-limited settings. *Lancet.* 2020;395(10233):1322–1325. 10.1016/S0140-6736(20)30798-4 32247324 PMC7270833

[13] Kochhar S Clarke E Izu A : Immunization in pregnancy safety surveillance in low and middle-income countries- field performance and validation of novel case definitions. *Vaccine.* 2019;37(22):2967–2974. 10.1016/j.vaccine.2019.03.074 31014963

[14] Law B Sturkenboom M : D2. 3 priority list of adverse events of special interest: COVID-19.Oslo, NO: CEPI.2020.

[15] Prevention CfDCa: Monitoring Systems for Pregnant People.Updated March 3, 2021.

[16] WHO: COVID-19 vaccines: safety surveillance manual.2020. Reference Source

[17] Stuurman AL Riera M Lamprianou S : Vaccine safety surveillance in pregnancy in low- and middle-income countries using GAIA case definitions: A feasibility assessment. *Vaccine.* 2018;36(45):6736–6743. 10.1016/j.vaccine.2018.09.033 30266486

[18] Pingray V : Using maternal and neonatal data collection systems for coronavirus disease 2019 (COVID-19) vaccines active safety surveillance in low- and middle-income countries: an international modified Delphi study - Supplementary materials.2021. 10.17605/OSF.IO/ZNS2D PMC11266593 39049963

[19] Aguilar MD Bernstein SJ Burnand B : The RAND/UCLA appropriateness method : user's manual.2001. Reference Source

[20] Lancet Commission on COVID-19 Vaccines and Therapeutics Task Force Members: Urgent needs of low-income and middle-income countries for COVID-19 vaccines and therapeutics. *Lancet.* 2021;397(10274):562–564. 10.1016/S0140-6736(21)00242-7 33516284 PMC7906712

[21] Grenham A Villafana T : Vaccine development and trials in low and lower-middle income countries: Key issues, advances and future opportunities. *Hum Vaccin Immunother.* 2017;13(9):2192–2199. 10.1080/21645515.2017.1356495 28758824 PMC5617553

[22] Hartmann K Pagliusi S Precioso A : Landscape analysis of pharmacovigilance and related practices among 34 vaccine manufacturers' from emerging countries. *Vaccine.* 2020;38(34):5490–5497. 10.1016/j.vaccine.2020.06.016 32591289 PMC7311355

[23] Groman D Higgins D Khan S : Lessons learned from the Advancing Maternal Immunization collaboration: identifying evidence gaps for informed respiratory syncytial virus maternal immunization decision-making [version 1; peer review: 2 approved]. *Gates Open Res.* 2019;3:1544. 10.12688/gatesopenres.13060.1 32025632 PMC6978845

[24] Bresee JS Lafond KE McCarron M : The partnership for influenza vaccine introduction (PIVI): Supporting influenza vaccine program development in low and middle-income countries through public-private partnerships. *Vaccine.* 2019;37(35):5089–5095. 10.1016/j.vaccine.2019.06.049 31288998 PMC6685526

[25] Gupta SB Coller BA Feinberg M : Unprecedented pace and partnerships: the story of and lessons learned from one Ebola vaccine program. *Expert Rev Vaccines.* 2018;17(10):913–923. 10.1080/14760584.2018.1527692 30269612

[26] Sobanjo-ter Meulen A Munoz FM Kaslow DC : Maternal interventions vigilance harmonization in low- and middle-income countries: Stakeholder meeting report; Amsterdam, May 1–2, 2018. *Vaccine.* 2019;37(20):2643–2650. 10.1016/j.vaccine.2019.03.060 30955981 PMC6546955

[27] Krubiner CB Faden RR Karron RA : Pregnant women & vaccines against emerging epidemic threats: ethics guidance for preparedness, research, and response. *Vaccine.* 2021;39(1):85–120. 10.1016/j.vaccine.2019.01.011 31060949 PMC7735377

[28] Ozawa S Grewal S Portnoy A : Funding gap for immunization across 94 low- and middle-income countries. *Vaccine.* 2016;34(50):6408–6416. 10.1016/j.vaccine.2016.09.036 28029541 PMC5142419

[29] Institute of Medicine, Committee on Ethical and Legal Issues Relating to the Inclusion of Women in Clinical Studies: Women and Health Research: Ethical and Legal Issues of Including Women in Clinical Studies: Volume I. In: Mastroianni AC, Faden R, Federman D, eds. National Academies Press (US), Copyright 1994 by the National Academy of Sciences. All rights reserved.;1994. 10.17226/2304

[30] van der Graaf R van der Zande ISE den Ruijter HM : Fair inclusion of pregnant women in clinical trials: an integrated scientific and ethical approach. *Trials.* 2018;19(1):78. 10.1186/s13063-017-2402-9 29378652 PMC5789693

[31] Brandon AR Shivakumar G Inrig SJ : Ethical Challenges in Designing, Conducting, and Reporting Research to Improve the Mental Health of Pregnant Women: The Voices of Investigators and IRB Members. *AJOB Empirical Bioethics.* 2014;5(2):25–43. 10.1080/23294515.2013.851128

[32] Blehar MC Spong C Grady C : Enrolling pregnant women: issues in clinical research. *Womens Health Issues.* 2013;23(1):e39–e45. 10.1016/j.whi.2012.10.003 23312713 PMC3547525

[33] Frew PM Saint-Victor DS Isaacs MB : Recruitment and retention of pregnant women into clinical research trials: an overview of challenges, facilitators, and best practices. *Clin Infect Dis.* 2014;59 Suppl 7(Suppl 7):S400–7. 10.1093/cid/ciu726 25425718 PMC4303058

[34] Payne P : Including Pregnant Women in Clinical Research: Practical Guidance for Institutional Review Boards. *Ethics Hum Res.* 2019;41(6):35–40. 10.1002/eahr.500036 31743630

