# Supplementary material for: Using maternal and neonatal data collection systems for coronavirus disease 2019 (COVID-19) vaccines active safety surveillance in low- and middle-income countries: an international modified Delphi study
Source: Gates Open Res. 2021 Jul 12;5:99. [Version 1] doi: 10.12688/gatesopenres.13305.1 (PMC11266593; doi:10.12688/gatesopenres.13305.1)
Supplement: Peer review history

# Reviewer report: Reviewer response for version 1

**DOI:** 10.21956/gatesopenres.14547.r30866

**Published:** 2024-07-23

**Authors:** Tariq Azim

**Affiliations:** 1 University of Limerick, Limerick, County Limerick, Ireland; 2 John Snow Incorporated (JSI) Research and Training Institute, Arlington, VA, USA; 3 Milken Institute School of Public Health, George Washington University, Washington, DC, USA

This is an important piece of work that can contribute to establishing a COVID vaccine surveillance system for pregnant women. The results are interesting and presenting the disagreement on the feasibility of setting such information system in LMICs is very important. The sample size was small and then there were drop outs. Though expected, this further reduced the number of participants. This should be mentioned in the limitation section.

 Some minor editing is required.

 Overall, this is a good study and ready to be indexed.

Is the work clearly and accurately presented and does it cite the current literature?

Yes

If applicable, is the statistical analysis and its interpretation appropriate?

Yes

Are all the source data underlying the results available to ensure full reproducibility?

Yes

Is the study design appropriate and is the work technically sound?

Yes

Are the conclusions drawn adequately supported by the results?

Yes

Are sufficient details of methods and analysis provided to allow replication by others?

Yes

Reviewer Expertise:

Health management information system; monitoring and evaluation; maternal and child health; tuberculosis

I confirm that I have read this submission and believe that I have an appropriate level of expertise to confirm that it is of an acceptable scientific standard.

# Reviewer report: Reviewer response for version 1

**DOI:** 10.21956/gatesopenres.14547.r32405

**Published:** 2024-07-23

**Authors:** Russell S. Kirby

**Affiliations:** 1 College of Public Health, University of South Florida, Tampa, FL, USA

The authors present the results of a modified Delphi study concerning the utility of existing maternal and neonatal data systems in LMICs for COVID-19 vaccine safety surveillance. The methods and approach seem sound, the only large concern being whether the sample of content experts is large enough to fully capture all issues and perspectives.

 Respondents provided generally congruent assessments, but also some concerns about the ability of existing data systems to be extended to the purpose of COVID-19 vaccine safety surveillance. Given the range of countries involved and the variation in their data capacity, it seems reasonable to anticipate that there would be no one-size-fits-all solution.

 But, in terms of how the study was designed and executed, it seems quite reasonable.  

 It would be interesting to learn about current vaccine safety surveillance practices in the LMICs, to better gauge whether existing systems are sufficient or could be modified for the more specific purpose of surveillance of vaccine use in pregnancy and among young children.

Is the work clearly and accurately presented and does it cite the current literature?

Yes

If applicable, is the statistical analysis and its interpretation appropriate?

Yes

Are all the source data underlying the results available to ensure full reproducibility?

Yes

Is the study design appropriate and is the work technically sound?

Yes

Are the conclusions drawn adequately supported by the results?

Yes

Are sufficient details of methods and analysis provided to allow replication by others?

Yes

Reviewer Expertise:

Population health science, perinatal and pediatric epidemiology, informatics, maternal and child health

I confirm that I have read this submission and believe that I have an appropriate level of expertise to confirm that it is of an acceptable scientific standard.

# Reviewer report: Reviewer response for version 1

**DOI:** 10.21956/gatesopenres.14547.r31575

**Published:** 2022-01-04

**Authors:** Waldemar A. Carlo

**Affiliations:** 1 Department of Pediatrics, University of Alabama at Birmingham, Birmingham, AL, USA

**Abstract:**

- In Results, the following sentence is incomplete: "A basic set of 14 maternal, neonatal, and vaccination-related variables". It seems these are extra words as the statement repeats in the sentence that follows.

**Introduction:**

- In the last paragraph of the Introduction section where it says “to build consensus about the use of existing maternal and neonatal data collection systems in LMICs for active safety surveillance of COVID-19 vaccines post-licensure, as well as the basic set of variables needed, it seems that post-emergency use authorization should be added. The terms post-licensure/post-authorization are inconsistently used.

**Methods:**

- The three-stage Delphi study was conducted between June 29th, 2020, and August 26th, 2020. This process ended over a year ago and before the vaccines got emergency use authorization. It is possible that some opinions will have changed in the interim year especially given that there have been so many publications on the topic of COVID in pregnancy plus the clinical differences in outcomes with the COVID variants. At this stage, would it be too difficult to add an additional stage in case opinions have changed?
- An important limitation of the study as a consequence of the early survey is that it was not known at the time of the survey the maternal and neonatal characteristics that would be most pertinent to collect. For example, microcephaly was ranked high but it is known now that it is not pertinent. The number of doses is likely to be important but these data were not ranked most likely as this has emerged as recent evidence has been reported. I think it is important to address in the Discussion the specific discrepancies between the maternal, neonatal, and vaccine variables selected by this process and what the current knowledge is.

**Safety surveillance:**

- Under the Panel section, the following sentence appears to be missing parts possibly before “as well as”: "During the recruitment process, as well as experts were selected based of their knowledge in maternal and perinatal health information systems for active safety surveillance."
- The review was conducted and reported in line with the standards of the COREQ statement for qualitative studies and STROBE checklist for observational studies. These guidelines are not specific for Delphi studies. The authors are encouraged to use the Guidance on Conducting and Reporting Delphi Studies (CREDES) guidelines in addition.

**Results:**

- It is stated that most participants were working at institutions conducting projects related to COVID-19 vaccines (13/16). The credential of “working at institutions conducting projects related to COVID-19 vaccines” does not describe well expertise in areas related to the study. It would be better to provide characteristics of the individuals rather than of the institutions where they work. The information about the characteristic of the individuals provided in the text does not match what is provided in Table 1.
- It would be important to give details of the number of panelists with expertise in the various areas mentioned in the Methods (“All the nominated experts had recognized international leadership in maternal immunization, vaccine safety, and maternal and neonatal health) as to what expertise was searched for in potential panel members. Indeed, a number of panelists meeting each of the three areas of international leadership would be important information to report, as this in part validates how the panelists met the planned criteria.
- There is too much emphasis on vaccine approval but it is likely that most studies will be performed initially under emergency use authorization. For example, in the section that addresses that the panel consistently disagreed (DI 2.45; 1.18; 1.79 for rounds 1 through 3, respectively) on the feasibility of existing maternal and neonatal data collection systems in LMICs to incorporate new changes before approval or licensure of novel COVID-19 vaccines so that adequate active safety surveillance can be performed amongst pregnant women (Table 3), should emergency use authorization not be included also?

**Discussion:**

- A major limitation that must be addressed is that the Delphi rounds were conducted at a time of limited specific knowledge about COVID vaccine-specific ideal collection data. While there is evolving knowledge on the topic, the knowledge has increased markedly since the last Delphi round so the long time gap from the last round to the present is a major limitation that is not addressed.
- It is essential to mention maternal, neonatal and vaccine-related data that could be considered to be added or deleted and differ from the data identified during the Delphi rounds.
- Many of the COVID references are dated (eg. MMWR 2020). For example, there are many missing references related to outcomes after COVID vaccination during pregnancy. The manuscript by Blakeway *et al. *(Am J Obstet Gynecol 2021 Aug 10;S0002-9378(21)00873-5. doi: 10.1016/j.ajog.2021.08.007. COVID-19 vaccination during pregnancy: coverage and safety) ^1^ should be quoted. Similarly, there are many related studies. A better review of the most current literature is needed.

Is the work clearly and accurately presented and does it cite the current literature?

No

If applicable, is the statistical analysis and its interpretation appropriate?

Partly

Are all the source data underlying the results available to ensure full reproducibility?

Partly

Is the study design appropriate and is the work technically sound?

Partly

Are the conclusions drawn adequately supported by the results?

No

Are sufficient details of methods and analysis provided to allow replication by others?

No

Reviewer Expertise:

Neonatology, maternal fetal medicine, research methods and protocol development, prematurity

I confirm that I have read this submission and believe that I have an appropriate level of expertise to confirm that it is of an acceptable scientific standard, however I have significant reservations, as outlined above.

## References

[1] : COVID-19 vaccination during pregnancy: coverage and safety. *American Journal of Obstetrics and Gynecology* .2021; 10.1016/j.ajog.2021.08.007 10.1016/j.ajog.2021.08.007 34389291 PMC8352848
